# Ocular Manifestations and Complications of Patent Foramen Ovale: A Narrative Review

**DOI:** 10.3390/jpm14070695

**Published:** 2024-06-27

**Authors:** Filippo Lixi, Luca Fazzini, Claudia Cannas, Roberta Montisci, Giuseppe Giannaccare

**Affiliations:** 1 Eye Clinic, Department of Surgical Sciences, University of Cagliari, 09124 Cagliari, Italy; f.lixi1@studenti.unica.it (F.L.); claudiacannas.94@gmail.com (C.C.); 2 Clinical Cardiology Unit, Department of Medical Sciences and Public Health, University of Cagliari, 09124 Cagliari, Italy; luca.fazzini10@gmail.com (L.F.); rmontisci@unica.it (R.M.)

**Keywords:** patent foramen ovale, eye, eye disorders, retinal artery occlusion, migraine with aura, impaired eye movement

## Abstract

Patent foramen ovale (PFO) is a prevalent congenital cardiac anomaly associated with a persistent opening between the atrial septum, allowing communication between the left and right atria. Despite often being asymptomatic, PFO can lead to various clinical presentations, including cryptogenic stroke and other embolic events. Transient visual disturbances, alterations in the visual field, migraine with aura, impaired eye movement and endogenous eye infections may prompt patients to seek ophthalmological consultation. Understanding these diverse clinical scenarios is crucial for early detection, appropriate management and mitigating the morbidity burden associated with PFO. This narrative review aims at examining the spectrum of clinical presentations of ocular pictures associated with PFO. The pathophysiology, diagnosis and treatment methods for PFO will be described, emphasizing the importance of a multidisciplinary approach involving ophthalmologists, cardiologists, neurologists and imaging specialists. In the future, prospective studies and clinical trials are warranted to provide further insights into the preventive role and optimal therapeutic strategies for managing PFO-related ocular complications, ultimately guiding clinical decision making and optimizing patient care.

## 1. Introduction

Patent foramen ovale (PFO) is a common congenital cardiac anomaly characterized by a persistent opening between the atrial septum, which allows for communication between the left and right atria [1]. After birth, changes in blood pressure lead to the closure of the foramen ovale in about 75% of newborns. As a result, its prevalence is estimated to be around 25–30% in the general population [2,3,4]. This defect creates a potential pathway for paradoxical embolism, allowing venous thrombi or other embolic material to bypass pulmonary circulation and enter systemic circulation, leading to deleterious clinical consequences [5].

Despite often being asymptomatic, PFO has been associated with various clinical presentations, ranging from cryptogenic stroke to different embolic events [5,6]. To highlight the impact of PFO, patients with cryptogenic stroke have a 2.9 times higher likelihood of having it compared to controls [7]. PFO may be implicated in approximately two-thirds of cryptogenic stroke cases and potentially up to 80% in younger patients [8]. In addition to the well-known and extensively studied complications documented in the literature, more potential clinical presentations have been linked to PFO. These presentations may encompass heterogeneous, less common, yet clinically relevant symptoms that may prompt patients to seek ophthalmic consultation. These symptoms could include transient visual disturbances, alterations in the visual field, migraine and other oculomotor signs that raise suspicion of possible PFO-related complications [9,10]. Several studies and case reports have described acute retinal ischemic events, including transient visual loss and retinal artery occlusions. Understanding the potential clinical manifestations of PFO is crucial for early detection and appropriate management, particularly considering the highly significant prevalence of this condition. Recognizing these manifestations early can facilitate prompt referral to cardiology, appropriate diagnostic work-up and consideration for available PFO closure treatment [11].

In this review, we will analyze the spectrum of clinical presentations associated with PFO, primarily focusing on manifestations that may drive patients to ophthalmological evaluation, also trying to highlight the importance of mitigating the morbidity burden associated with PFO, given its high prevalence in the general population.

## 2. Materials and Methods

We utilized the PubMed medical database for our search. The term “patent foramen ovale” was combined with the words “eye”, “eye disorders”, “eye diseases”, “retina”, “ophthalmology”, “eye palsy”, “migraine with aura”. The text keywords were chosen using information from pertinent bibliographies and the latest literature. The earliest publishing date of the search was set for January 1990, and it ended in March 2024.

Only English-language articles and reviews were analyzed, even though no language limits were placed on the searches. Moreover, manual searches were conducted within initial findings to identify further bibliographic references.

A total of 801 full-text articles were identified on PubMed, 679 of which were excluded after the first screening. The remaining 122 articles were evaluated for eligibility. After a full-text evaluation, 73 papers were retained as relevant articles and were used in this review.

## 3. Results

### 3.1. Patent Foramen Ovale: Pathophysiology, Diagnosis and Screening

PFO is derived from embryological folding misplacement. Briefly, the septum primum originates from the upper part of the atrium. The septum secundum forms through a folding of the atrial walls, creating the ostium secundum, which serves as a passage for oxygenated blood to flow from right to left during fetal life. A PFO occurs when the septum primum and septum secundum remain unjoined near the anterior and superior edge of the oval fossa. PFO size in adults is heterogeneous, ranging from 1 mm to 15–19 mm [4]. Of note, larger size and specific morphologies have been shown to correlate with enhanced risk of cerebrovascular accidents [12,13].

Usually, the diagnosis of PFO is made incidentally during routine imaging studies or after the patient presents with a potential clinical manifestation, prompting further imaging evaluation. The diagnosis is pursued through multimodality ultrasound imaging, including transthoracic echocardiography (TTE), transesophageal echocardiography (TEE) and transcranial Doppler using aerated saline as a contrast agent. TTE is often the initial screening tool, providing valuable information about the cardiac structure and function. However, its sensitivity in detecting PFO may be limited due to technical factors and the position of the septum, probably leading to minimal shunting miss [14,15]. Conversely, TEE offers higher sensitivity and specificity that reaches 100% when a contrast agent is used [16,17], allowing for closer visualization of the interatrial septum and providing detailed images of the fossa ovalis properly describing the PFO size and morphology [17]. It is particularly useful in cases in which TTE results are inconclusive or when a higher level of detail is required. Additionally, TEE enables the assessment of associated cardiac anomalies and facilitates the guidance of percutaneous closure procedures. Complementing echocardiographic techniques, by monitoring cerebral blood flow, transcranial Doppler can detect microembolic signals arising from right-to-left shunting through the PFO, especially when contrast injection is performed before Valsalva maneuver [18]. Its sensitivity was shown to be heterogeneous compared to intracardiac imaging [19,20,21,22]. However, these imaging modalities together play a crucial role in the comprehensive evaluation and management of patients with suspected PFO.

Screening for a PFO should be selectively conducted among individuals who have recently experienced clinical events potentially suggestive of PFO presence and who stand to benefit from PFO treatment. TEE is indicated in patients with a higher likelihood of having a PFO, for example, when assessing young individuals with unexplained strokes or any other plausible symptom linked to PFO; for patients with a lower probability of PFO, contrast TTE or transcranial Doppler may serve as reasonable initial diagnostic methods.

### 3.2. PFO and Acute Retinal Ischemic Events

Transient monocular vision loss (TMVL)/amaurosis fugax (AF), central retinal artery occlusion (CRAO), branch retinal artery occlusion (BRAO) and cilioretinal artery occlusion (CLRAO) are acute retinal ischemic events manifesting as sudden, painless monocular visual losses with different degrees of severity. The occlusion of the retinal artery can be transient or permanent and may result in impaired blood flow in the ocular circulation, which is associated with a high cardiovascular morbidity and mortality risk [23].

TMVL/AF is a condition characterized by a transient episode of monocular blindness that typically lasts for seconds to minutes. It occurs due to a temporary interruption of blood flow at the level of the ophthalmic artery or central retinal artery and its branches. The most common cause of TMVL/AF in the elderly population is embolism, characterized by a small clot or debris traveling through the bloodstream and blocking a blood vessel. These emboli can originate from various sites, including the carotid arteries, heart or other blood vessels [24]. Although in younger people, TMVL/AF is often associated with temporary spasm at the level of ocular circulation, monocular TMVL/AF may be the consequence of cardiogenic emboli secondary to cardiac malformations. Accordingly, in a recent cohort study, 15% of patients with TMVL/AF were found to have a PFO [25].

**Figure 1:**
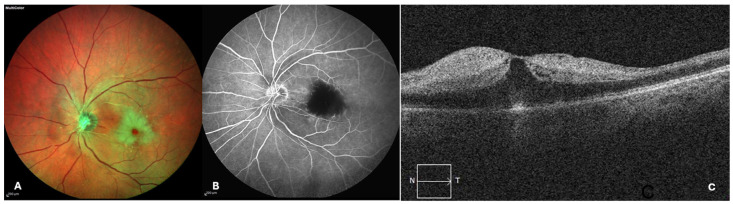
Retinal ischemic events related to PFO: (**A**) Multimodal imaging of a CRAO. On fundus photography, the retina at the posterior pole appears pale and opaque due to the ischemia; a distinctive cherry-red spot is visible at the fovea, which remains red due to the underlying choroidal circulation. The retinal arteries are thin and narrowed. (**B**) Fluorescein angiography reveals a hypofluorescent macular spot secondary to the ischemia of retinal layers. (**C**) The latter is confirmed by optical coherence tomography (OCT), which shows hyper-reflective and edematous inner retinal layers.

CRAO is a disease in which the main artery supplying blood to the retina becomes occluded. It manifests as an acute loss of vision, typically resulting in visual acuity of 20/400 or poorer; its incidence is 1 in 100,000 individuals, with a mean age of presentation of 60 years (Figure 1A–C) [26].

BRAO is a disorder in which one of the branches of the central retinal artery becomes blocked, leading to restricted blood flow to a portion of the retina; its incidence is closer to 5 per 100,000 persons per year [27]. The symptoms of BRAO can vary depending on the severity and location of the occlusion, but usually, the prognosis is better than in CRAO, resulting in a localized scotoma or in a sectoral visual field defect. Both of these ischemic disorders can be linked to PFO [28].

Another distinct clinical entity is the CLRAO. The cilioretinal artery arises from the short posterior ciliary vessels, and its prevalence ranges from 6.9% to 49.5%. It may play a role in providing additional blood supply to the macular region of the retina, thus preserving central vision, in cases where the main retinal vasculature is compromised, such as in CRAO [29]. CLRAO can be a consequence of a hemodynamic blockage of the artery caused by a rise in intraluminal pressure in retinal vasculature exceeding the level of perfusion pressure, or it can be secondary to embolism or thrombosis, or it can recognize an arteritic etiology. Symptoms of CLRAO may include sudden blurring, scotoma or distortion of central vision. Since CLRAO has been linked to PFO through an embolic mechanism, the presence of an underlying cardiac defect should be excluded in young people presenting this disorder [28,30,31].

The diagnosis of acute retinal ischemic events is clinical and based on different possible morphologic findings detectable on fundus examination, such as retinal afferent pupillary defect (RAPD), retinal whitening, macular cherry-red spot and intra-arterial emboli. Retinal fluorescein angiography, optical coherence tomography (OCT) and OCT angiography (OCTA) can confirm the diagnosis, showing delayed arterial filling, retinal ischemia and hyper-reflective inner retinal layers [31,32,33]. Therapeutical approaches, including anterior chamber paracentesis, drug-lowering intraocular pressure and ocular massage, are often useless [34].

Despite being rare among younger patients, retinal arterial obstructions are described to be linked to underlying cardiac abnormalities in 45% of individuals below the age of 45 [35]. Numerous reports have detailed the connection between PFO and retinal ischemic disorders [25,28,30,31,32,33,36,37,38,39,40,41,42,43,44,45,46,47,48,49,50,51] (Table 1). In a cohort of 18 patients diagnosed with either central or branch retinal artery occlusion, TEE was conducted to probe potential underlying cardiac or aortic disorders as sources of retinal emboli. The investigation unveiled that 17% of the patients exhibited a PFO [48]. Fekri and colleagues analyzed 23 cases of retinal ischemic events in the presence of PFO, revealing a comparable incidence of BRAO and CRAO. Most cases were under 50 years old (78.3%), and roughly 50% of patients underwent uncomplicated PFO closure. TEE exhibited higher sensitivity than TTE in diagnosing the underlying cardiac disease (71.4% versus 28.6%) [28]. Similar findings were reported by Wieder et al., demonstrating that TEE detected PFO in 85.7% of cases compared to 14.3% with TTE in the context of ischemic retinal disorders in patients with a mean age of 42.4 years [32].

While the exact pathogenesis of retinal arterial blockage in individuals with PFO remains incompletely understood, several mechanisms have been proposed. These include emboli formation within the atrial septum, transient arrhythmias leading to thrombus formation and paradoxical emboli originating from the peripheral venous system, which reach the head circulation occluding the ophthalmic artery and its branches [33,37].

Once diagnosed, considering PFO closure may be advisable to reduce the risk of additional ischemic vasculo-occlusive events [28,33,39,41,44,45,46].

Moreover, in addition to well-known risk factors like smoke and hypertension, an association with conditions such as internal carotid artery (ICA) hypoplasia, filler injections and pregnancy has also been described in patients with PFO developing retinal ischemic events [26,31,32,36,41,49,50,51].

ICA is one of the most important blood sources to the head, and its hypoplasia is rare. Many cases of abnormal development of ICA are asymptomatic, thanks to the compensatory action of collateral circulation. However, Zhu and co-authors reported a case of an apparently healthy woman who developed an AF due to the presence of a concomitant PFO in association with ICA hypoplasia that resulted in an embolism at the level of the retinal artery [49].

Facial filler with hyaluronic acid (HA) has previously been linked to visual loss. The retrograde flow mechanism is widely recognized as the primary explanation for sudden vision loss following HA injection for facial filler procedures. This phenomenon occurs when the injected filler material inadvertently enters a blood vessel, leading to its backward movement against the natural blood flow direction until it reaches the ocular circulation [52]. Nevertheless, in a recent report, a young woman presented multiple retinal artery ischemic events occurring in a peculiar pattern. Indeed, the patient’s visual loss presented in two stages, rather than directly after injection, and was associated with dyspnea. Hence, the authors speculated that the HA entered systemic venous circulation, reached the right heart, passed directly into the left heart through a PFO and then caused retinal artery occlusion [50].

Various physiological adaptations, encompassing complex cardiovascular, coagulative, hormonal and immunological changes, occur during pregnancy and can trigger vascular occlusive events. The concomitant presence of PFO increases the risk of ocular thromboembolic events, as demonstrated by retinal vascular occlusion episodes described in two different reports. In these situations, OCTA, as a dye-free, non-invasive tool, represents a valuable option to diagnose and analyze the retinal perfusion status in pregnancy [31,51].

Therefore, PFO should be considered in cases of young patients who develop unexplained acute retinal ischemic events.

### 3.3. PFO and Migraine with Aura

Migraine is a neurological disorder characterized by recurrent, severe headaches, often accompanied by other symptoms, such as nausea, vomiting, sensitivity to light and visual disorder. Migraine attacks can cause significant pain and discomfort, sometimes lasting for hours to days, and can greatly disrupt daily life activities. Migraine with aura is a type of migraine characterized by specific neurological and visual symptoms, known as “aura”, which usually occurs before the headache phase. The aura symptoms can vary widely among individuals but commonly include visual disturbances, such as flashing lights, blind spots or zigzag patterns which are usually bilateral. Other sensory disorders like tingling sensations in the face or hands, speech difficulties and confusion can be present. These symptoms develop gradually over a few minutes and last for about 20–60 min before the headache phase begins [53]. Multifactorial causative mechanisms, such as genetic, environmental and neurological factors, are recognized to be involved in migraine. Different triggers include stress, bright lights, certain foods, changes in sleep patterns, hormonal variations, sensory stimuli, weather changes and strong odors [53].

Migraine prevalence varies across different populations and regions, with a higher incidence among females [54]. The association between PFO and migraine has been widely described in the literature [10,55,56] (Table 2). Specifically, the relationship between PFO and migraine with aura was first described in a study carried out by Del Sette and colleagues. Among 80 migraineurs with aura, with an average age of 37.24 years, 36 individuals (45%) were found to have a cardiac right-to-left shunt [55]. Furthermore, migraine with aura appears to be more prevalent in individuals with PFO, and the latter is more common in migraineurs with aura than in the general population. Accordingly, a meta-analysis conducted by Schwedt et al. demonstrated a higher prevalence of PFO among migraine patients with aura (40.9–72.0%) compared to migraineurs without aura (16.2–33.7%). Moreover, the prevalence of migraine in individuals with PFO varied from 22.3% to 64.3% [10].

The pathophysiology of migraine with aura in patients with PFO remains uncertain. Various vasoactive substances, including serotonin or 5-hydroxytryptamine, typically processed through the pulmonary circulation, may bypass this route via the PFO, potentially triggering migraines. Additionally, small emboli passing through the PFO into the arterial system could lead to microinfarctions or cortical spreading depression, precipitating migraine attacks. Furthermore, focal areas of reduced blood flow near the ischemic threshold in the occipital regions may lead to visual symptoms during the aura episode [56,57].

Although multiple studies have investigated the effect of closing PFO on migraine, the therapeutic effect of this surgical procedure is still controversial. The MIST trial found no significant difference between patients who underwent PFO closure and those who did not [58]. On the other hand, the PRIMA trial did not demonstrate a reduction in its primary endpoint based on decreasing migraine days [59], while the PREMIUM trial showed a statistically significant decrease in headache days but failed to achieve a reduction in its primary outcome, defined as a responder rate with a 50% decrease in migraine attacks [60]. Conversely, other reports showed a significant decrease in the frequency of migraine attacks following PFO closure in patients with migraine with aura [61,62,63,64,65,66]. Specifically, an overall reduction in the frequency of migraine attacks per day and a decrease in the number of headache days were observed over one month [65,66]. Moreover, in patients whose migraine episodes were with aura, there was a decrease in migraine attacks following PFO closure compared to control groups [63].

Considering that visual symptoms usually precede the headache, ophthalmologists play an important role in diagnosing migraine with aura and differentiating it from other ophthalmic conditions that may present with similar symptoms, such as retinal ischemia or retinal detachment. Additionally, ophthalmologists may collaborate with neurologists in the management of migraine with aura, providing guidance on lifestyle modifications and strategies to manage migraine triggers (e.g., bright lights, stress and irregular sleep patterns) that may exacerbate symptoms. Moreover, they may prescribe medications to alleviate symptoms or recommend specialized examinations, such as visual field testing or neuroimaging studies, to further evaluate the underlying cause of symptoms [67].

### 3.4. PFO and Impaired Eye Movement

**Figure 2:**
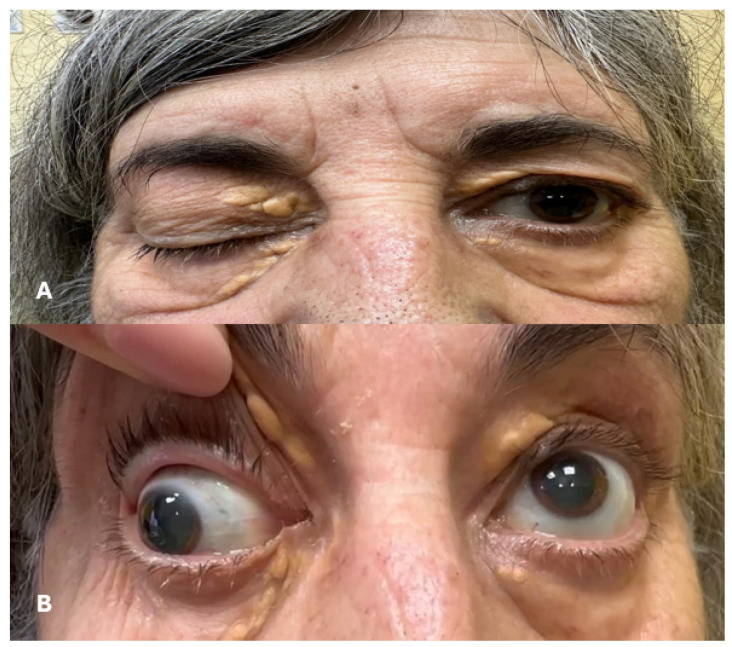
Impaired eye movement related to PFO: (**A**) Right eye third nerve palsy presenting a complete ptosis. (**B**) After eyelid elevation, the right eye appears to be positioned downward and outward, with inability to adduce, infraduce or overduce. The pupil is dilated with slow reaction.

Eye movements are coordinated motions of the eyes, which allow perception of the surrounding space, the tracking of objects and focusing on specific targets. These movements are controlled by a complex system, involving muscles, nerves and various brain regions. Oculomotor disfunctions are characterized by the weakness or paralysis of the muscles within or surrounding the eye, resulting in impaired ocular action or control (Figure 2A,B). These can be caused by different factors, including nerve damage, trauma, inflammation, underlying systemic disorders or ischemic strokes. Injuries at the level of brain visual-related areas can impair the oculomotor system, resulting in several alterations in coordination, visual acuity and visual field orientation [68].

The association between PFO and impaired eye movement is rare, and only few cases have been described [69,70,71,72,73] (Table 3). Cerebral lesions, involving the midbrain and other vision areas, were considered the consequence of paradoxical emboli resulting from the direct pass in the vascular system and in the cerebral circulation through the right–left shunt [69,70,71,72,73]. Internuclear ophthalmoplegia (IO)—a disorder characterized by a lesion of medial longitudinal fasciculus resulting in an ipsilesional adduction deficit with a nystagmus of the abducting eye—is often secondary to brain ischemia. A 39-year-old woman presented bilateral ptosis and IO associated with PFO diagnosed with TEE. After undergoing PFO closure and starting aspirin therapy for secondary prevention, the patient remained clinically stable over a one-year follow-up. [69]. A previously healthy 16-year-old girl experienced an IO secondary to a lesion of the left midbrain at the level of quadrigeminal lamina. Six months after aspirin therapy and PFO closure, her cerebral lesions and her eye movements were restored [70]. In addition, Zhuang and colleagues reported a case of a 55-year-old male who experienced a complete peripheral facial palsy and horizontal gaze palsy after Valsalva maneuver caused by paradoxical embolization from PFO. After 6 months and surgical closure, the symptoms improved [71]. Conversely, a 61-year-old man who had a severe embolic event involving the posterior cerebral artery territory, resulting in left hemiparesis, dysarthria, bilateral ptosis, and impaired eye movement on both sides, experienced only partial improvement after anticoagulant therapy. [72]. Khan described a case of an acute pupil-sparing complete third nerve palsy (complete ptosis, moderate hypotropia, large exotropia and no supraduction/infraduction/adduction) in a healthy young woman with PFO. After PFO closure, the neurological recovery was complete, except for diplopia and relatively comitant hypotropia, which responded well to conventional strabismus surgery [71]. Thus, despite only a few cases having been reported, PFO should be considered in cases of impaired eye movement secondary to embolic stroke in apparently healthy people [69,70,71,72,73].

### 3.5. PFO and Endogenous Eye Infections

**Figure 3:**
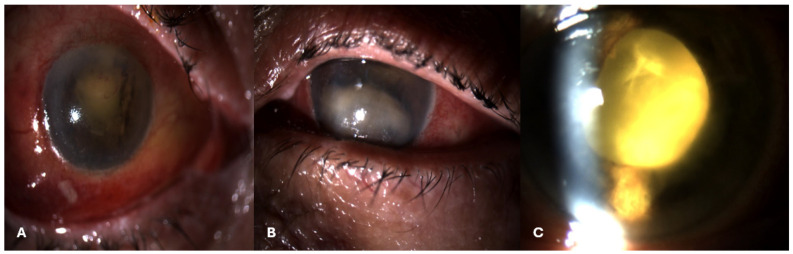
Endogenous eye infection related to PFO: (**A**–**C**) Slit lamp images of endophthalmitis showing conjunctival redness, purulent discharge, chemosis, hypopyon and fibrin in the pupillary field.

Endophthalmitis refers to inflammation and infection of the fluids and tissues inside the eye and can result in devastating sight-threatening and systemic complications (Figure 3A–C). It can recognize an exogenous or endogenous origin. Most cases are of exogenous origin, typically arising from microbes entering the eye through an external source like penetrating trauma, intraocular surgery or corneal infection. Conversely, endogenous endophthalmitis results from the spread of bacterial or fungal infections from a distant site in the body, typically through the bloodstream [74]. In this type of endophthalmitis, the damage occurs mainly due to a septic embolus entering the posterior segment vasculature and acting as a trigger for the dissemination of micro-organisms into the surrounding tissues. After passing the blood–ocular barrier, the infection extends from the retina and choroid into the vitreous cavity, and subsequently, to the anterior chamber of the eye [75]. The common sources of infection leading to endogenous endophthalmitis include bacterial endocarditis, intravenous drug use and certain systemic infections like candidiasis or tuberculosis [74].

Rodrigues et al. described a case of endogenous endophthalmitis that developed as the first manifestation of right-sided endocarditis in a patient with an undiagnosed PFO [76]. A 66-year-old female with chronic renal failure presenting fever and eye endophthalmitis was treated with systemic antibiotics and enucleation. However, since a persistent systemic infection with positive blood cultures was observed, adjunctive analyses, including TEE, were performed, and a right endocarditis with a PFO was diagnosed [76]. Hence, even though paradoxical embolization through a PFO from right-sided endocarditis is rare [77], the presence of an interatrial defect caused ocular infection in this case. Therefore, an underlying cardiac defect should be taken into account in cases of endogenous endophthalmitis, where the source of primary infection is unidentified.

## 4. Discussion

**Figure 4:**
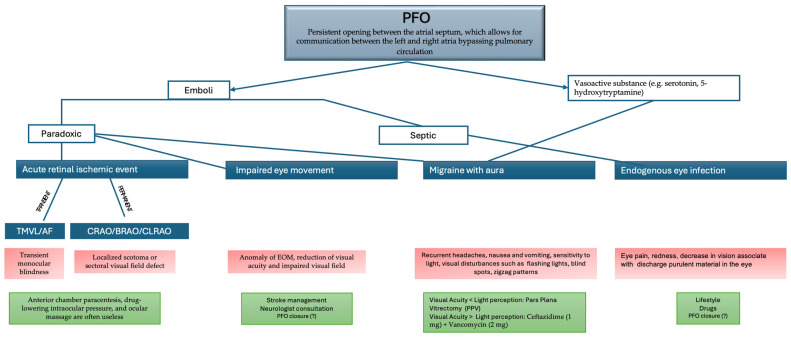
The diagram schematically illustrates the mechanism by which ophthalmic complications occur due to PFO. The symptoms of each condition are presented in the red squares, while the green squares show the possible management strategies.

The spectrum of clinical presentations associated with PFO encompasses a wide array of manifestations, including ophthalmological conditions like retinal artery occlusions, migraine with aura, impaired eye movements and endogenous infections (Figure 4).

The management of arterial occlusion remains a subject of ongoing debate. In the acute phase, the foremost goal for the ophthalmologist is to promptly restore retinal blood flow. Prolonged hypoxia has been demonstrated to lead to irreversible visual damage, underscoring the urgency of this intervention. Moving into the subacute phase, the ophthalmologist’s focus shifts to assessing the patient in order to ascertain the underlying causative mechanism. This evaluation is crucial for preventing further ocular or systemic complications [34]. Despite the lack of clinical trials, the associations between PFO and acute retinal ischemic events have been described in multiple reports. According to retinal and ophthalmic artery occlusions guidelines provided by the American Academy of Ophthalmology, conditions like ophthalmic artery occlusion, CRAO and BRAO can potentially indicate life-threatening situations [78]. Therefore, particularly in cases where the initial examination does not reveal a causative mechanism for embolic disease, considering the significant risk of ischemic stroke associated with ocular arterial occlusions, ophthalmologists must refer patients to a stroke center for multidisciplinary medical evaluation, including echocardiography and other imaging analyses.

Ophthalmologists frequently serve as the initial specialists to evaluate individuals experiencing visual symptoms related to migraines. The exact pathophysiological mechanisms linking PFO to migraine with aura remain uncertain. However, the higher prevalence of PFO among migraineurs with aura compared to the general population was shown in several studies, suggesting a relationship between these two entities. Although PFO closure has shown contrasting results in the management of migraine, considering the low complication rate of the procedure, surgical intervention, as secondary prevention, represents a valid option.

Managing ocular palsies typically involves several approaches, depending on the underlying cause, which need to be identified, and on the severity of the condition. Impaired eye movement in the presence of PFO is rarely reported. Nevertheless, in cases of cryptogenic embolic strokes affecting visual motility-related brain regions, the clinical suspicion of PFO should be raised, especially in young patients.

When suspecting endogenous endophthalmitis, an accurate diagnostic evaluation is crucial to identify a potential hematogenous spread of micro-organisms from a distant source in the body. It is worth noting that the presence of PFO may elevate the risk of paradoxical embolization. Indeed, particularly in patients with predisposing factors, such as bacterial endocarditis, endogenous ocular infection could be secondary to the presence of an undetected communication between the two atria. Despite ongoing debates, the consensus on the therapeutic approach to these conditions remains elusive. However, according to the guidelines from the European Society of Cataract and Refractive Surgery, pars plana vitrectomy appears to offer the most favorable visual outcomes in cases where visual acuity is limited to light perception. For patients with visual acuity exceeding light perception, the therapeutic strategies remain uncertain. Evidence suggests that combining the same antibiotics via both intravitreal and systemic routes is the most effective approach. Administering ceftazidime (1 mg) and vancomycin (2 mg) separately is considered the gold standard [79].

In summary, after diagnosing an ocular embolic event, ophthalmologists should ideally require further investigation, including a TTE or a TEE, by echocardiographic imaging specialists. Once a PFO is diagnosed, potential interventional therapies should be considered, including PFO closure and medical therapy. The meta-analysis by Vukadinović et al. indicated that percutaneous PFO closure significantly reduces the risk of ischemic recurrences compared to medical therapy, especially in cases of large or moderate shunts [80]. Similarly, Turc et al. stated that PFO closure is superior to antithrombotic therapy in preventing stroke recurrence after cryptogenic stroke despite an increased risk of atrial fibrillation after the surgical procedure [81]. Conversely, Liu and colleagues reported that, compared with drug therapy, PFO closure reduces the risk of recurrent stroke and the incidence of serious bleeding without increasing the risk of new-onset atrial fibrillation or atrial flutter [82].

Hence, while the direct involvement of ophthalmologists in diagnosing PFO itself may be limited, their expertise in assessing ocular health and recognizing subtle signs of systemic diseases is invaluable. The correlation between ocular signs and symptoms and systemic diseases has been well documented. Recently, the interest in identifying ocular risk factors or biomarkers for diagnosing or excluding systemic diseases has significantly increased, leading to the emergence of oculomics. By examining the eye’s structure and function, oculomics aims to provide insights into a patient’s overall health, identifying the potential risk factors for diseases beyond the eye [83]. In this context, the deep-learning algorithm RetiCAC, which uses retinal photographs to predict the presence of coronary artery calcium (CAC), has demonstrated improved cardiovascular risk stratification compared to traditional clinical parameters [84].

Nonetheless, collaboration with other specialists like cardiologists and neurologists still remains essential for ensuring timely diagnosis, comprehensive evaluation and appropriate treatment selection.

## 5. Conclusions

It should be noted that PFO is a highly common condition that can cause severe ocular complications. This review analyzed the diverse clinical scenarios in which PFO may be implicated, shedding light on the importance of early recognition and multidisciplinary management in optimizing patient care. Ophthalmologists, as the first healthcare professionals to encounter patients presenting with ocular symptoms and signs, upon identifying suspicious findings, should be aware of this possible subtle entity and promptly refer patients to appropriate specialists.

## 6. Future Directions

Given the limited number of reports on certain ocular disorders associated with PFO, future prospective studies and clinical trials are warranted to provide further insights into the preventive role and optimal therapeutic strategies for managing these conditions. Large-scale, multicenter studies with long-term follow-up periods would help elucidate the natural history of PFO-related ocular complications. Additionally, effective research comparing different treatment modalities and their impact on clinical outcomes would be valuable for guiding clinical decision making and optimizing patient care.

## Figures and Tables

**Table 1:** Summary of the studies showing retinal ischemic events in patients with PFO.

Author (Year)	Reference	Type of Study	Population	Outcomes
Sverdlichenko et al. (2024)	[25]	Prospective cohort study	20 cases under 45 years of age with TMVL (4 men and 16 women)	All patients included were referred for neuroimaging and cardiac investigations. Two of the thirteen patients who completed echocardiography had PFO (15%).
Fekri et al. (2024)	[28]	Case-study-based review	23 cases of CRAO (n = 10), BRAO (n = 10) and CILRAO (n = 3) with PFO	A total of 78.3% of the cases were under 50 years of age. A high RoPE score (≥7, suggestive of paradoxical embolism via PFO) was estimated for 71.4% of patients. TEE exhibited higher sensitivity than TTE in diagnosing the underlying cardiac disease (71.4% versus 28.6%). Approximately half of the patients underwent percutaneous closure of the PFO with no complications.
Jurgens et al. (2022)	[31]	Review and case report	1 case	A pregnant woman developed cilioretinal artery occlusion of thromboembolic origin associated with PFO. OCTA enabled risk-free imaging of retinal vessel perfusion during pregnancy.
Wieder et al. (2021)	[32]	Review and case report	1 case	A 43- year-old man presented with CRAO in the setting of PFO. TEE appeared more sensitive than TTE in diagnosing PFO (85.7% versus 14.3%).
Chatziralli et al. (2015)	[33]	Case report	1 case	A 35-year-old man presented with a left inferior BRAO. Cardiologic examination with TEE revealed a right-to-left shunt across PFO. The patient developed no other embolic event at the 9-month follow-up, having undergone an operation for PFO repair.
Inatomi et al. (2001)	[36]	Retrospective study	22 cases	A total of 22 patients with retinal artery occlusion (RAO) were analyzed. TEE findings were abnormal in 13 (59%) of the 22 patients. PFO was observed in 9% of patients.
Tayyab et al. (2023)	[37]	Case report	1 case	A teenage girl presented with CRAO in the right eye, which was accompanied by frontal headaches and vertigo. Investigations to rule out hematologic, vascular and cardiac causes were performed. TEE revealed PFO as the cause of this cryptogenic stroke.
Ho and Spaide (2007)	[38]	Case report	1 case	A healthy 15-year-old boy developed a CRAO with cilioretinal artery sparing after a fractured clavicle. Initially, no abnormalities were found after extensive systemic examinations. Subsequently, PFO was revealed by TEE, and the patient was scheduled for cardiac surgery.
Gabrielian et al. (2009)	[39]	Case report	1 case	A healthy 17-year-old male presented with sudden, painless loss of vision in his right eye while playing basketball. Fundus examination and fluorescein angiography confirmed a retinal artery occlusion. The hematological/infectious work-up, including TTE, was negative. However, TEE showed PFO. The patient underwent a successful percutaneous femoral catheterization to close the defect.
Nakagawa et al. (2004)	[40]	Case report	1 case	A 43-year-old woman presented a bilateral CRAO within one month. The patient was treated with urokinase and with an anticoagulant. The corrected VA recovered completely in the right eye and partially in the left eye. After extensive examination, TEE revealed a right-to-left shunt through PFO. The patient was treated with anticoagulants and has suffered no recurrence of CRAO.
Ascuitto et al. (2021)	[41]	Case report	1 case	A patient presented with painless, sudden visual loss in his left eye. He was diagnosed with CRAO, likely from a paradoxical embolus associated with an atrial septal aneurysm containing PFO. The patient underwent successful percutaneous closure of the PFO without complication.
Shoeibi et al. (2013)	[42]	Case report	1 case	A 29-year-old female patient presented a BRAO. The patient was treated with oral acetazolamide, topical timolol, ocular massage and anterior chamber paracentesis. The visual field defect partially recovered. After an extensive work-up, a small-sized PFO was detected by TEE. Low-dose aspirin therapy was initiated, and over the subsequent two years, no other embolic event occurred.
Wisotsky et al. (1993)	[43]	Case report	2 cases	A 28-year-old man and a 19-year-old woman presented BRAO. In each patient, PFO was demonstrated via transesophageal echocardiography after a transthoracic study disclosed no abnormalities.
Liu et al. (2017)	[44]	Case report	1 case	A 21-year-old man presented with an eight-day history of a blurry patch of vision in the right eye. A BRAO was diagnosed after fundus and OCT examinations. A TTE bubble contrast study showed a large right-to-left shunt at the atrial level. Percutaneous closure of the PFO was undertaken. Normal visual acuity after 12 months was recovered.
Grudzińska et al. (2021)	[45]	Case report	1 case	A 33-year-old Caucasian man presented a superior nasal loss of visual field and central scotoma in his left eye. A BRAO was diagnosed. The patient underwent standard TTE without any pathological findings. TEE revealed a marked leak through PFO, and cardiologists decided on foramen ovale closure.
Sheth et al. (2009)	[46]	Case report	1 case	An 18-year-old male claimed sudden-onset left visual loss. Ophthalmological examination revealed an afferent pupillary defect secondary to left hemiretinal artery occlusion. A subsequent cardiology review with TEE revealed PFO, which was closed surgically.
Sabanis et al. (2020)	[47]	Case report	1 case	A patient with no significant comorbidities experienced a paradoxical thromboembolic episode of CRAO. After a comprehensive examination, PFO was diagnosed after a bubble contrast TTE.
Kramer et al. (2001)	[48]	Retrospective observational case series	18 cases	A total of 18 patients who were diagnosed with retinal artery occlusion (7 CRAO, 11 BRAO) were analyzed. Cardiac or thoracic aortic pathologic conditions, which were a possible source of the retinal emboli, were detected by TEE in 13 of the 18 patients (72%). They included aortic arch atheroma (n = 7), mitral annulus calcification (n = 4), left atrial appendage thrombus (n = 2), valvular abnormalities (n = 5), left atrial smoke (n = 3) and PFO (n = 3). TEE showed higher sensitivity than TTE in detecting the cardiac emboli source.
Zhu et al. (2021)	[49]	Case report	1 case	A 46-year-old woman presented an amaurosis fugax lasting 20 min and a persistent visual alteration in her left eye. Fundus examination did not reveal significant abnormalities. Fundus fluorescein angiography showed a long arm–retina circulation time. Imaging examinations revealed bilateral internal carotid artery (ICA) hypoplasia and a variation in the circle of Willis. Furthermore, TEE showed the presence of a PFO and a cardiogenic embolic event. Aspirin 100 mg/day was given to the patient, and PFO occlusion surgery was recommended.
Si et al. (2023)	[50]	Review and case report	1 case	A 40-year-old woman reported a gradual decline in visual acuity, with visual field defects that had developed in two stages after HA injection as facial filler into the nasal root. Multiple retinal artery occlusions were diagnosed. PFO was detected by TEE. The patient was treated with intravenous dexamethasone and cobamamide, as well as extracorporeal counterpulsation therapy. Visual acuity improved from counting fingers at 30 cm to 20/133 within 3 days.
Pipolo et al. (2023)	[51]	Case report	1 case	A 28-year-old woman presented at the clinic 20 h after an acute visual field defect in the right eye. At that time, she was 18 weeks pregnant and had no report of complications in her previous pregnancy. After ophthalmological evaluation, BRAO was diagnosed. A transcranial Doppler with contrast indicated a right-to-left intracardiac shunt, confirmed by the presence of PFO at the TEE. Thrombophilic conditions were excluded. Enoxaparin 1 mg/kg was started and kept until the delivery. Then, a surgical closure of PFO was programmed.

**Table 2:** Summary of the studies showing correlation between migraine and PFO.

Author (Year)	Reference	Type of Study	Population	Outcomes
Schwedt et al. (2008)	[10]	Systematic review	18 studies, 1517 patients	The aim of this study was to examine the prevalence of migraine in patients with PFO, the prevalence of PFO in migraineurs and the effect of PFO closure on migraine. The estimated strength of association between PFO and migraine, reflected by summary odds ratios (ORs), was 5.13 [95% confidence interval (CI) 4.67, 5.59], and between PFO and migraine with aura, the OR was 3.21 (95% CI 2.38, 4.17). The association between migraine and PFO was OR 2.54 (95% CI 2.01, 3.08). Six studies on PFO closure suggested improvement in migraine. The low-to-moderate grade of evidence from observational studies supports an apparent association between PFO and migraine. PFO closure seemed to affect migraine patterns favorably.
Del Sette et al. (2008)	[55]	Prospective study	87 patients	The objective of this study was to clarify the relationship between right-to-left shunt (RLS) and white matter lesions (WMLs) in patients with migraine with aura (MA). Among 80 migraineurs with aura, with an average age of 37.24 years, 36 individuals (45%) were found to have a cardiac right-to-left shunt. The comparison of T2-weighted MRI scans revealed no significant difference in the number of lesions or lesion loads between patients with and without RLS. In conclusion, RLS does not increase the likelihood of finding WMLs in migraineurs.
Liu et al. (2020)	[56]	Narrative review	99 articles	In this article, the potential pathophysiological mechanisms of migraine, the clinical symptoms of migraine with PFO and the clinical features of PFO with migraine were described. The incidence of PFO in migraine patients is higher than that in the general population, suggesting that PFO and migraine may be risk factors for each other, but more research is needed to confirm this speculation. An increasing number of studies have found that migraines with aura are more closely associated with PFO, and the presence of right-to-left shunt (RLS) increased the likelihood of aura attacks. The evidence supports a “dose–response” relationship between migraine and PFO, although more work needs to be conducted in terms of patient selection as well as the inclusion of an antiplatelet control group for PFO closure interventions to uncover possible beneficial results in clinical trials.
Wilmshurst (2018)	[57]	Randomized controlled trial	147 patients	The Migraine Intervention with STARFlex Technology (MIST) trial was a randomized double-blind trial in patients with severe intractable migraine with aura and moderate–large PFO that compared the implantation of STARFlex devices with the intention of closing their PFO versus a sham procedure. The results showed no significant difference in migraine outcomes between those receiving the STARFlex implant and those in the sham group.
Dowson et al. (2008)	[58]	Randomized controlled trial	432 patients	The aim of this study was to investigate whether closing patent foramen ovale (PFO) could effectively treat migraine with aura. Patients were randomly assigned to transcatheter PFO closure with the STARFlex implant or to a sham procedure. No significant difference was observed in the primary endpoint of migraine headache cessation between the implant and sham groups.
Mattle et al. (2016)	[59]	Randomized controlled trial	107 patients	The Percutaneous Closure of Patent Foramen Ovale In Migraine with Aura (PRIMA) study aimed to evaluate the efficacy of percutaneous patent foramen ovale (PFO) closure in reducing migraine symptoms in patients diagnosed with migraine with aura. Patients were divided in two groups and treated with a PFO closure or medical treatment. Despite the safety of the procedure, PFO closure did not reduce overall monthly migraine days.
Tobis et al. (2017)	[60]	Randomized controlled trial	230 patients	The Prospective, Randomized Investigation to Evaluate Incidence of Headache Reduction in Subjects With Migraine and PFO Using the AMPLATZER PFO Occluder to Medical Management (PREMIUM) study investigated migraine reduction in subjects with persistent migraines and PFO. It compared medical therapy alone to therapy combined with PFO closure using the Amplatzer device. The trial found no superior reduction in migraine attacks with PFO closure compared to sham control. However, it did show a significant decrease in headache days among subjects with or without aura following PFO closure.
Giardini et al. (2006)	[61]	Prospective study	38 patients	This study evaluated the long-term outcomes of transcatheter patent foramen ovale (PFO) closure on migraine headache with aura (MHA) and recurrent stroke risk. Among 38 patients followed for at least 3 years after the procedure, 92% reported complete resolution of MHA after an average of 4.9 years. Recurrent stroke occurred in 5.3% of cases within 5 years, predominantly within the first 15 months after the procedure. Overall, the MIDAS scores significantly decreased (from 38.6 ± 26.3 to 4.4 ± 5.1, *p* < 0.0001) following intervention.
Yan et al. (2024)	[62]	Retrospective study	78 patients	This study aimed to investigate the therapeutic efficacy of patent foramen ovale (PFO) closure in migraine patients with a massive right-to-left shunt (RLS). The study included 51 patients without white matter lesions (WMLs) (control group, CG) and 27 patients with white matter lesions (WMLs) (observation group, OG). PFO closure proved effective and safe in treating migraine patients with RLS. However, for those with WMLs, clinical attention should be directed toward the treatment of WMLs.
Elbadawi et al. (2018)	[63]	Meta-analysis	3 studies, 484 patients	The analysis included three key studies: the MIST, PRIMA and PREMIUM trials. The primary outcome demonstrated a significant reduction in monthly migraine attacks, favoring PFO closure over control groups (*p* = 0.01). Although complete resolution of migraine attacks showed a trend favoring PFO closure, the difference did not achieve statistical significance. In patients with predominant aura migraine attacks, PFO closure demonstrated a significant reduction in migraine attacks. No significant differences in responders’ rate between PFO closure and control groups were found.
Shi et al. (2017)	[64]	Meta-analysis and systematic review	8 articles, 546 patients	The study investigated how the closure of patent foramen ovale (PFO) affected migraine in 546 patients, distinguishing between those with migraine with aura (MA) and migraine without aura (MwoA). Following PFO closure, migraine symptoms improved in 81% of MA patients and 63% of MwoA patients. The benefits of PFO closure were significantly greater for patients with MA compared to patients with MwoA (*p* = 0.03).
Zhang et al. (2022)	[65]	Meta-analysis	3 RCTs, 1 pooled study, 8 case series, 1165 patients	The aim of the study was to evaluate the impact of patent foramen ovale (PFO) closure on migraine. The findings revealed a statistically significant reduction in both monthly migraine attacks and migraine days, along with a substantial decrease in migraine frequency and severity in patients undergoing PFO closure, particularly those with migraines accompanied by aura (MA). The meta-analysis supports PFO closure as a potentially effective and safe therapeutic option for reducing the migraine burden, particularly in patients with MA.
Zhang et al. (2021)	[66]	Meta-analysis	3 RCTs, 4 observation studies, 887 patients	The meta-analysis findings indicate that PFO closure leads to a substantial reduction in both monthly migraine attacks and migraine days per month compared to control groups. This effect was particularly pronounced in patients with migraine accompanied by aura, where there was a notable increase in the rate of complete cessation of migraine attacks following the procedure. These results underscore the potential of PFO closure as an effective therapeutic option for managing migraine, especially in cases where traditional treatments have been insufficient.

**Table 3:** Summary of the articles showing correlation between impaired eye movement and PFO.

Author (Year)	Reference	Type of Study	Population	Outcomes
Xie et al. (2022)	[69]	Case report	1 case	A 39-year-old woman presented with sudden bilateral ptosis and diplopia exacerbated by leftward gaze, persisting for 3 days. Neurological examination and MRI confirmed acute midbrain infarction. The tests excluded other stroke causes but confirmed a large right-to-left shunt via PFO. She underwent PFO closure and received aspirin.
Mazurkiewicz-Bełdzińska et al. (2015)	[70]	Case report	1 case	A healthy 16-year-old girl had sudden diplopia and dizziness following a headache. MRI confirmed left midbrain ischemia with left internuclear ophthalmoplegia. PFO was discovered by TTE and TEE. Medical treatment led to full recovery of neurological deficits in two weeks. Six months after aspirin therapy and PFO closure, her cerebral lesions and her eye movements were restored.
Zhuang et al. (2021)	[71]	Case report	1 case	A 55-year-old man presented with sudden bilateral gaze palsy, upbeat nystagmus and facial paralysis. MRI revealed new pontine infarctions linked to PFO with significant shunt. He was treated with rivaroxaban and underwent PFO closure, resolving his symptoms. A follow-up MRI at 6 months showed no new lesions.
Takahashi et al. (2018)	[72]	Case report	1 case	A 61-year-old heavy smoker presented with sudden vision distortion and left leg weakness, revealing multiple brain infarctions correlated with PFO. Treatment included anticoagulants and antiplatelets, but severe residual deficits persisted after 12 weeks.
Khan (2012)	[73]	Case report	1 case	A healthy 23-year-old woman experienced sudden right third nerve palsy and facial weakness. MRI showed midbrain infarction linked to a large PFO. Surgical closure resolved the symptoms; strabismus surgery corrected residual right hypotropia, ensuring stable vision postoperatively.
